# The Tri-State Medical Association of Alabama, Georgia and Tennessee

**Published:** 1892-08

**Authors:** 


THE Tri-State Medical Society of Alabama, Georgia and Tennessee will hold its fourth annual meeting in Chattanooga, beginning Tuesday, October 25th, 1892. The meeting will last three days That it will be a success, is assured by the fact that Dr. W. E. B. Davis, the president of this young and vigorous society, is working very hard for the success of the next meeting.

The membership includes many from States other than those included in the title. Papers have been promised by the following: Drs. W. E. B. Davis, Rome, Ga.; I. N. Love, St. Louis, Mo.; J. B. Cowan, Tullahoma, Tenn.; E. B. Ward, Selma, Ala.; J. M. Head, Zebulon, Ga.; John L. Howell, Knoxville, Tenn.; C. S. Briggs, Nashville, Tenn.; J. M. Masters, Knoxville, Tenn.; Richard Douglass, Nashville, Tenn.

